# ESBL/pAmpC-producing *Escherichia coli* and *Klebsiella pneumoniae* carriage among veterinary healthcare workers in the Netherlands

**DOI:** 10.1186/s13756-021-01012-8

**Published:** 2021-10-19

**Authors:** Anouk P. Meijs, Esther F. Gijsbers, Paul D. Hengeveld, Cindy M. Dierikx, Sabine C. de Greeff, Engeline van Duijkeren

**Affiliations:** Centre for Infectious Disease Control, National Institute for Public Health and the Environment (RIVM), PO Box 1, 3720 BA Bilthoven, The Netherlands

**Keywords:** ESBL, pAmpC, *Escherichia coli*, *Klebsiella pneumoniae*, Antibiotic resistance, Carriage, Veterinary clinics, Veterinary healthcare workers

## Abstract

**Background:**

Animals are a reservoir for ESBL/pAmpC-producing *Escherichia coli/Klebsiella pneumoniae* (ESBL-E/K). We investigated the association between occupational contact with different types of animals and the prevalence of ESBL-E/K carriage among veterinary healthcare workers, assessed molecular characteristics of ESBL-E/K, and followed-up on the ESBL-E/K carriage status of participants and their household members.

**Methods:**

Participants completed a questionnaire about their contact with animals at work and at home, health status, travel behaviour and hygiene, and sent in a faecal sample which was tested for the presence of ESBL-E/K. Resistance genes were typed using PCR and sequencing*.* ESBL-E/K positive participants and their household members were followed up after 6 months. Risk factors were analysed using multivariable logistic regression methods.

**Results:**

The prevalence of ESBL-E/K carriage was 9.8% (47/482; 95%CI 7.4–12.7). The most frequently occurring ESBL genes were *bla*_CTX-M-15_, *bla*_CTX-M-14_ and *bla*_DHA-1_. The predominant sequence type was ST131. None of the occupation related factors, such as contact with specific animal species, were significantly associated with ESBL-E/K carriage, whereas travel to Africa, Asia or Latin America in the past 6 months (OR 4.4), and stomach/bowel complaints in the past 4 weeks (OR 2.2) were. Sixteen of 33 initially ESBL-E/K positive participants (48.5%) tested positive again 6 months later, in 14 persons the same ESBL gene and *E. coli* ST was found. Four of 23 (17.4%) household members carried ESBL-E/K, in three persons this was the same ESBL gene and *E. coli* ST as in the veterinary healthcare worker.

**Conclusions:**

Despite the absence of specific occupation related risk factors, ESBL-E/K carriage in veterinary healthcare workers was high compared to the prevalence in the general Dutch population (5%). This indicates that occupational contact with animals is a potential source of ESBL-E/K for the population at large.

**Supplementary Information:**

The online version contains supplementary material available at 10.1186/s13756-021-01012-8.

## Introduction

Extended Spectrum Beta-Lactamase (ESBL) and plasmid-mediated AmpC (pAmpC)-producing Enterobacterales (ESBL-E), including *Escherichia coli* and *Klebsiella pneumoniae* (ESBL-E/K), were initially associated with infections in the healthcare setting [1]. During the last decades, ESBL-E were established in the general population as well [2]. Humans are exposed to these bacteria through animals, food products, the environment and human-to-human contact, the latter presumably being the largest contributor to the spread of ESBL-E [3]. In the Netherlands, the prevalence of ESBL-E carriage in the population at large is approximately 5% [4, 5].

ESBL-E is also frequently found in companion animals and livestock [6–9], and higher prevalences of ESBL-E have been found in persons working on farms in close contact with poultry [8, 10]. Furthermore, similar ESBL genes were found in pig and poultry farmers and their animals, indicating transmission between animals and humans [8–10]. ESBL-E transmission via contact with companion animals and livestock other than broilers and pigs seems to occur less frequently [11–13].

Veterinary healthcare workers might be at an increased risk of acquiring ESBL-producing bacteria due to their close contact with large numbers of animals, and their exposure to antibiotics in their daily practice environment. In the present study, we aim to explore the association between occupational contact with different types of animals and the prevalence of ESBL-E/K carriage. The objectives of this study were, (1) to investigate the prevalence of carriage of ESBL-E/K in Dutch veterinary healthcare workers, (2) to characterize these ESBL-E/K and their resistance genes, (3) to assess risk factors for ESBL-E/K carriage within veterinary health care workers, and (4) to follow-up on the ESBL-E/K carriage status of a subgroup of participants and their household members 6 months later.

## Methods

### Study design

This study is part of the Antibiotic Resistance in Dutch Veterinary healthcare workers study (Dutch acronym: AREND), in which the presence of ESBL-E/K, colistin resistant *Escherichia coli* or *Klebsiella pneumoniae* (ColR-E/K) and *Clostridioides difficile* was determined in persons working in veterinary healthcare. The medical ethical committee of the University Medical Center Utrecht reviewed this study and granted it an official exemption for approval under the WMO (number 18-389/C). All participants signed an informed consent form. Participants were recruited in 2018 at the annual Dutch veterinary conference, via articles in newsletters and journals for veterinarians, and by information about the study sent directly to veterinary clinics. Criteria for inclusion were age 18 years or older and working in veterinary care. Enrolment of participants was distributed over the years between August 2018 and March 2019. Persons working in the same clinic were assigned to participate in different months to avoid the possibility of clustering. Participants were invited to fill in a web-based questionnaire about their contact with animals at work and at home, hygiene, health and medication use and leisure activities such as travel behaviour. They received a package to collect a faecal sample at home and were asked to send it to our laboratory by regular mail on the day of sampling. Individual culture results were reported to participants who indicated that they wanted this on their informed consent form.

All participants with positive culture results for ESBL-E/K received an invitation and an informed consent form to take part in the longitudinal component of the study. The longitudinal part comprised of a second faecal sampling approximately 6 months after the initial sample (between March 2019 and October 2019) and a short additional questionnaire, with questions on changes in occupation, contact with animals, health and medication use and leisure activities in the preceding 6 months. Furthermore, household members (≥ 18 years of age) of participants were invited to participate as well. Their participation included sending in a faecal sample and a questionnaire about their relation to the veterinary healthcare worker, occupation, contact with animals, health and medication use and leisure activities, including travel.

### Microbiology and genotyping

Upon arrival at the laboratory, the faecal samples were either processed the same day or stored at 4 °C for up to 2 days. Samples were cultured on Brilliance *E. coli*/coliform Selective Agar (Oxoid) with and without 1 mg/L cefotaxime (BECSA^+^ and BECSA^−^) (Sigma) and incubated overnight at 37 °C to determine the presence of ESBL-E/K. In addition, a cotton swab with faecal material was incubated overnight at 37 °C in 2 mL of Luria Bertani broth (MP Biomedicals) supplemented with 1 mg/L cefotaxime. The following day, 10 µL of the enrichment broth was streaked on BECSA^+^ and incubated overnight at 37 °C. If plates showed suspected growth of ESBL-E/K (after direct plating and/or enrichment), three colonies per sample (blue, pink and/or purple) were selected for further testing (see Meijs et al. for a more detailed description) [14]. Pink coloured colonies were further analysed to determine bacterial species using Matrix Assisted Laser Desorption/Ionisation Time-Of-Flight Mass Spectrometry (MALDI-TOF MS) (Bruker). Presumptive positive ESBL-E/K isolates were characterized by multilocus sequence typing (MLST) [15, 16], and ESBL-genes were typed using polymerase chain reaction (PCR) and sequencing; see Additional file 1: Table S1.

### Statistical analyses

The confidence interval (CI) for the prevalence of ESBL-E/K was calculated using the Wilson score. Risk factors for ESBL-E/K carriage were determined by logistic regression models. Results are presented as odds ratio’s with 95% CIs. First, univariate analyses were performed for potential risk factors being sex, age, birth country, children attending day-care, urbanization level, season of participation, type of profession in animal healthcare, animal contact at work, occupation of household members, keeping pets/farm animals for a hobby, animal contact at home, hospitalization, use of proton-pump inhibitors (PPIs) and antibiotics, medication use, stomach/bowel complaints, travel history and leisure activities, diet, and kitchen and toilet hygiene. Variables with a *p*-value < 0.20 in univariate analysis and sex and age were selected for the multivariable logistic regression model. The multivariable model was reduced using a backward selection method until all variables in the model reached statistical significance (*p*-value < 0.05). Analyses were performed using SAS V. 9.4 (SAS Institute Inc., Cary, NC, USA).

## Results

**Fig. 1:**
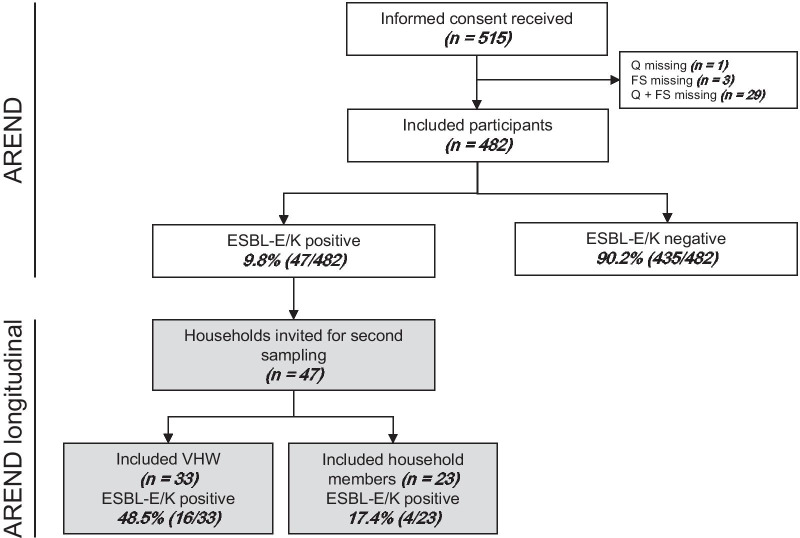
Flow diagram of participating veterinary healthcare workers and their household members. AREND: antibiotic resistance in Dutch veterinary healthcare workers study; ESBL-E/K: extended-spectrum beta-lactamase or pAmpC-producing *Escherichia coli/Klebsiella pneumoniae*; FS: faecal sample; Q: questionnaire; VHW: veterinary healthcare worker

Of the 515 veterinary healthcare workers (veterinary workers) who signed the informed consent form, 482 (93.6%) returned both the faecal sample and questionnaire (Fig. 1). The median age of the participants was 38 years (min 20; max 70 years) and 84.9% were female. They were employed as either veterinarian (46.9%), veterinary technician (45.6%; including animal physiotherapists) or veterinary assistant (7.5%; including animal caretakers) (Table 1). Veterinary assistants, who more frequently perform administrative tasks, registered fewer animal contact hours per week compared with the other two groups. Furthermore, animal-related work activities such as performing consultations and surgical procedures differed between the professions. Veterinarians more frequently worked with livestock and horses compared with the other two groups and performed animal-related tasks such as home or farm visits more often. Other differences between the professions are shown in Table 1.

**Table 1:** Occupational characteristics of veterinarians, veterinary technicians and veterinary assistants

	Profession		
	Veterinarian n = 226 (46.9%)	Veterinary technician^a^ n = 220 (45.6%)	Veterinary assistant^b^ n = 36 (7.5%)
	n (%)	n (%)	n (%)
*Occupational animal contact*			
No. of years working in animal health care (median; IQR)	13 (6–22)	10.5 (5–18.5)	13 (8.5–25)
No. animal contact hours per week (median; IQR)	25 (20–30)	20 (10–28)	14.5 (5–27.5)
Frequent animal contact with^c^			
Companion animals	189 (83.6)	216 (98.2)	30 (83.3)
Dogs	185 (81.9)	214 (97.3)	27 (75.0)
Cats	181 (80.1)	208 (94.6)	28 (77.8)
Rabbits/guinea pigs/hamsters	154 (68.1)	145 (65.9)	16 (44.4)
Mice/rats	21 (9.3)	12 (5.5)	3 (8.3)
Birds	22 (9.7)	21 (9.6)	3 (8.3)
Livestock	73 (32.3)	31 (14.1)	6 (16.7)
Cattle	51 (22.6)	19 (8.6)	5 (13.9)
Pigs	14 (6.2)	3 (1.4)	2 (5.6)
Chicken	22 (9.7)	15 (6.8)	3 (8.3)
Other poultry	3 (1.3)	5 (2.3)	2 (5.6)
Sheep	34 (15.0)	13 (5.9)	4 (11.1)
Goats	25 (11.1)	10 (4.6)	4 (11.1)
Equines	42 (18.6)	30 (13.6)	6 (16.7)
Frequent animal contact with companion animals only	139 (61.5)	168 (76.4)	25 (69.4)
*Animal-related work activities*^*c*^			
Activities with companion animals			
Consultations	177 (78.3)	178 (80.9)	24 (66.7)
Home visits	75 (33.2)	10 (4.6)	0 (0)
Surgical procedures	152 (67.3)	150 (68.2)	19 (52.8)
Dental cleaning/care	126 (55.8)	139 (63.2)	18 (50.0)
Cleaning animal housing	84 (37.2)	198 (90.0)	25 (69.4)
Shaving/grooming	76 (33.6)	123 (55.9)	14 (38.9)
Activities with livestock			
Farm/home visits	61 (27.0)	2 (0.9)	0 (0)
Surgical procedures	41 (18.1)	1 (0.5)	0 (0)
Cleaning out stables	0 (0)	3 (1.4)	1 (2.8)
Activities with equines			
Outpatient clinic	6 (2.7)	2 (0.9)	0 (0)
Farm/home visits	33 (14.6)	2 (0.9)	0 (0)
Surgical procedures	6 (2.7)	1 (0.5)	0 (0)
Dental cleaning/care	7 (3.1)	2 (0.9)	0 (0)
Cleaning out stables	4 (1.8)	3 (1.4)	0 (0)
Brushing/grooming	4 (1.8)	4 (1.8)	0 (0)
Farm visits total (last 4 weeks)	65 (28.8)	6 (2.7)	1 (2.8)
Cattle farms	48 (21.2)	3 (1.4)	1 (2.8)
Beef cattle	33 (14.6)	1 (0.5)	0 (0)
Dairy cattle	48 (21.2)	3 (1.4)	1 (2.8)
Poultry farms	8 (3.5)	0 (0)	0 (0)
Broilers	5 (2.2)	0 (0)	0 (0)
Laying hens	5 (2.2)	0 (0)	0 (0)
Pig farms	20 (8.9)	0 (0)	1 (2.8)
Porkers	13 (5.8)	0 (0)	1 (2.8)
Meat pigs	15 (6.6)	0 (0)	0 (0)
Sheep farms	36 (15.9)	0 (0)	0 (0)
Goat farms	29 (12.8)	0 (0)	0(0)

IQR: interquartile range

^a^Including animal physiotherapists

^b^Including animal caretakers

^c^Weekly or more often

### Prevalence, sequence types and ESBL/pAmpC resistance genes

Forty-seven of the 482 participating veterinary workers were carriers of ESBL-E/K (prevalence 9.8%; 95% CI 7.4–12.7). One person (0.2%) carried a pAmpC-producing *K. pneumoniae (bla*_DHA-1_ in a new sequence type, see footnote of Table 2 for the allelic profile), in all other participants the ESBL/pAmpC genes were in *E. coli*. A total of 10 different ESBL/pAmpC genes were found. *bla*_CTX-M-15_ (n = 26) was the most common one, followed by *bla*_CTX-M-14_ (n = 7) and *bla*_DHA-1_ (n = 4). The most frequently found sequence types were ST131 (n = 9), ST38 (n = 5) and ST69 (n = 5). Table 2 shows that *bla*_CTX-M-15_ was the most common ESBL gene in both participants working with companion animals only and participants working with livestock, horses or a combination of horses, livestock and/or companion animals. All four *bla*_DHA-1_ were found in persons working with companion animals only. In five participants multiple ESBL-genes and/or *E. coli* STs were found. No clustering was observed of ESBL/pAmpC gene and sequence type combinations in participants working in the same location.

**Table 2:** ESBL/pAmpC gene types and *E. coli* and *K. pneumoniae* sequence types in veterinary healthcare workers

ESBL/pAmpC gene	Veterinary workers who work with companion animals only^a^ (*n* = *33*)		Veterinary workers who work with livestock, horses or a combination of horses, livestock and/or companion animals^a^ (*n* = *14*)	
	n (%)	ST (no. isolated)^c^	n (%)	ST (no. isolated)^d^
*bla*_CTX-M-15_	16 (48.5)	43 (1), 46 (1), 48 (1), 69 (1), 131 (4), 226 (1), 405 (1), 550 (1), 656 (1), 678 (1), 1193 (1), 1954 (1), 6438 (1), new (1)	9 (64.3)	10 (3), 95 (1), 131 (2), 394 (1), 442 (1), 1193 (1), 4684 (1)
*bla*_CTX-M-14_	6 (18.2)	38 (4), 93 (1), 744 (1)	1 (7.1)	69 (1)
*bla*_DHA-1_	4 (12.1)	10 (1), 349 (2), Kpn new (1)^e^	0 (0)	
*bla*_CTX-M-27/174_^b^	3 (9.1)	38 (1), 131 (2)	0 (0)	
*bla*_CTX-M-1_	2 (6.1)	69 (1), 349 (1)	0 (0)	
*bla*_CTX-M-55_	1 (3.0)	117 (1)	1 (7.1)	744 (1)
*bla*_CTX-M-15_ and *bla*_CMY-2_	1 (3.0)	167 (1)	0 (0)	
*bla*_CMY-2_	1 (3.0)	69 (1)	0 (0)	
*bla*_CTX-M-9_	0 (0)		1 (7.1)	131 (1)
*bla*_CTX-M-32_	0 (0)		1 (7.1)	48 (1), 64 (1)
*bla*_CTX-M-65_	0 (0)		1 (7.1)	69 (1)

ESBL: extended-spectrum beta-lactamase; Kpn, *Klebsiella pneumoniae;* pAmpC: plasmid-mediated AmpC; ST: sequence type

One sequence type belongs to *K. pneumoniae* (ST new with pAmpC gene *bla*_DHA-1_), all other sequence types belong to *E. coli*

^a^Based on frequency of contact (at least once a week)

^b^With the primers used no distinction could be made between *bla*_CTX-M-27_ and *bla*_CTX-M-174_

^c^In the isolates of one person two different ESBL-genes and STs were found (*bla*_CTX-M-14_ in ST93 and *bla*_CTX-M-15_ in ST678), and in the isolates of another person the same ESBL gene was found in two different STs (*bla*_CTX-M-15_ in ST6438 and ST1954)

^d^In the isolates of two persons the same ESBL gene was found in two different STs (person 1: *bla*_CTX-M-15_ in ST10 and ST4684; person 2: *bla*_CTX-M-32_ in ST48 and ST68)

^e^Allelic profile: gapA, allele 17; infB, allele 19; mdh, allele 188; pgi, allele 20; phoE, new allele (best match with allele 545 with 1 SNP (^322^G → ^322^A)); rpoB, allele 18; tonB, allele 197. This profile is most closely related to ST2637 and ST5563

### Risk factors

Statistically significant risk factors for ESBL-E/K carriage in univariate analysis were: very high urbanization level (≥ 2500 addresses/km^2^), ADHD medication use, Crohn’s disease, stomach and/or bowel complaints in the last 4 weeks, travel to Africa, Asia or Latin America in the last 6 months and swimming in salt water in the last 6 months (indicated in bold in Table 3). In multivariate analysis, only travel to Africa, Asia or Latin America (OR 4.41; 95% CI 2.11–9.19) and stomach and/or bowel complaints (OR 2.18; 95% CI 1.17–4.06) remained statistically significant. Crohn’s disease was not included in the multivariate analysis, since only four participants had Crohn’s disease, of whom two where ESBL-E/K positive. None of the occupation related factors, such as contact with specific types of animals (companion animals, livestock, horses) and profession (veterinarian, technician, assistant) were significantly associated with ESBL-E/K carriage.

**Table 3:** Assessment of risk factors of ESBL/pAmpC-producing *E. coli*/*K. pneumoniae* in veterinary healthcare workers

Determinant	ESBL-E/K prevalence	ESBL-E/K status n (%)		Univariable OR (95% CI)	Multivariable OR (95% CI)
		Negative (n = 435)	Positive (n = 47)		
	%	n (%)	n (%)		
Sex					
Male	12.3	64 (14.7)	9 (19.2)	1.37 (0.63–2.98)	
Female	9.3	371 (85.3)	38 (80.9)	Ref	
Age, years (median; IQR)	–	38 (31–48)	35 (29–45)	0.98 (0.96–1.01)	
Born in the Netherlands	9.6	426 (97.9)	45 (95.7)	0.48 (0.10–2.27)	
Has children (< 4 years of age) attending day-care	7.4	63 (14.5)	5 (10.6)	0.70 (0.27–1.85)	
Urbanisation level					
Very high (≥ 2500 Addresses/km^2^)	17.5	47 (10.8)	10 (21.3)	**2.31 (1.01–5.28)**^**e**^	
High/moderate (1000–2500 addresses/km^2^)	8.6	181 (41.6)	17 (36.2)	1.02 (0.51–2.02)	
Low/very low (< 1000 addresses/km^2^)	8.4	206 (47.4)	19 (40.4)	Ref	
Season of participation					
Spring	10.6	59 (13.6)	7 (14.9)	1.56 (0.52–4.67)	
Summer	10.3	35 (8.1)	4 (8.5)	1.50 (0.41–5.45)	
Autumn	10.4	249 (57.2)	29 (61.7)	1.53 (0.65–3.62)	
Winter	7.1	92 (21.2)	7 (14.9)	Ref	
*Occupational animal contact*					
Profession					
Veterinarian	11.5	200 (46.0)	26 (55.3)	1.55 (0.82–2.95)^d^	
Veterinary technician^a^	7.7	203 (46.7)	17 (36.2)	Ref	
Veterinary assistant^b^	11.1	32 (7.4)	4 (8.5)	1 49 (0.47–4.72)	
No. of animal contact hours per week (median; IQR)	–	20 (15–30)	25 (15–30)	1.00 (0.98–1.03)	
Frequent contact with companion animals^c^	9.7	393 (90.3)	42 (89.4)	0.90 (0.34–2.39)	
Frequent contact with livestock^c^	10.9	98 (22.5)	12 (25.5)	1.18 (0.59–2.36)	
Frequent contact equines^c^	7.7	72 (16.6)	6 (12.8)	0.74 (0.30–1.80)	
Animal contact with (last 4 weeks)					
Dogs	9.8	377 (86.7)	41 (87.2)	1.05 (0.43–2.59)	
Cats	9.7	371 (85.3)	40 (85.1)	0.99 (0.42–2.30)	
Rabbits/Guinea pigs/hamsters	9.1	299 (68.7)	30 (63.8)	0.80 (0.43–1.51)	
Rats/mice	10.4	60 (13.8)	7 (14.9)	1.09 (0.47–2.55)	
Birds	7.6	110 (25.3)	9 (19.2)	0.70 (0.33–1.49)	
Cattle	11.1	72 (16.6)	9 (19.2)	1.19 (0.55–2.58)	
Sheep	8.8	62 (14.3)	6 (12.8)	0.88 (0.36–2.16)	
Goats	9.1	50 (11.5)	5 (10.6)	0.92 (0.35–2.43)	
Chicken	9.5	76 (17.5)	8 (17.0)	0.97 (0.44–2.16)	
Other poultry	8.3	11 (2.5)	1 (2.1)	0.84 (0.11–6.64)	
Pigs	9.4	29 (6.7)	3 (6.4)	0.96 (0.28–3.26)	
Horses	6.4	88 (20.2)	6 (12.8)	0.58 (0.24–1.40)	
Reptiles	12.5	7 (1.6)	1 (2.1)	1.33 (0.16–11.04)	
Farm visits total (last 4 weeks)	8.3	66 (15.2)	6 (12.8)	0.82 (0.33–2.00)	
Cattle farms	7.7	48 (11.0)	4 (8.5)	0.75 (0.26–2.18)	
Poultry farms (including chicken)	12.5	7 (1.6)	1 (2.1)	1.33 (0.16–11.04)	
Pig farms	4.8	20 (4.6)	1 (2.1)	0.45 (0.06–3.44)	
Sheep farms	5.6	34 (7.8)	2 (4.3)	0.52 (0.12–2.26)	
Goat farms	10.3	26 (6.0)	3 (6.4)	1.07 (0.31–3.69)	
Hand washing frequency after patient contact					
(Almost) always	10.2	289 (66.4)	33 (70.2)	Ref	
Regularly/sometimes	8.7	137 (31.5)	13 (27.7)	0.83 (0.42–1.63)	
(Almost) never	16.7	5 (1.2)	1 (2.1)	1.75 (0.20–15.45)	
*Occupation of household member*					
Profession with animal contact	10.3	70 (16.1)	8 (17.0)	1.07 (0.48–2.39)	
Farmer/farm employee	9.1	10 (2.3)	1 (2.1)	0.92 (0.12–7.38)	
Veterinarian	7.5	37 (8.5)	3 (6.4)	0.73 (0.22–2.48)	
Veterinary assistant/technician	11.1	8 (1.8)	1 (2.1)	1.16 (0.14–9.49)	
Healthcare professional	9.4	29 (6.7)	3 (6.4)	0.96 (0.28–3.26)	
Physician	9.1	10 (2.3)	1 (2.1)	0.92 (0.12–7.38)	
Nurse	33.3	4 (0.9)	2 (4.3)	4.79 (0.85–26.88)^d^	
*Animal contact at home*					
Owning a pet or hobby farm animal	10.1	275 (63.2)	31 (66.0)	1.13 (0.60–2.13)	
Owning dog(s)	7.4	163 (37.5)	13 (27.7)	0.64 (0.33–1.24)^d^	
Owning cats(s)	9.8	166 (38.2)	18 (38.3)	1.01 (0.54–1.87)	
Owning rabbit(s)/Guinea pig(s)/hamster(s)	5.1	75 (17.2)	4 (8.5)	0.45 (0.16–1.28)^d^	
Owning rat(s)/mouse/mice	14.3	6 (1.4)	1 (2.1)	1.55 (0.18–13.19)	
Owning bird(s)	8.8	31 (7.1)	3 (6.4)	0.89 (0.26–3.03)	
Owning cow(s)	14.3	6 (1.4)	1 (2.1)	1.55 (0.18–13.19)	
Owning sheep	9.1	20 (4.6)	2 (4.3)	0.92 (0.21–4.08)	
Owning chicken	9.1	70 (16.1)	7 (14.9)	0.91 (0.39–2.12)	
Owning horse(s)	9.5	57 (13.1)	6 (12.8)	0.97 (0.39–2.39)	
Owns companion animal that eats raw meat	6.3	30 (6.9)	2 (4.3)	0.56 (0.13–2.48)	
*Health and medication use*					
Hospitalized in Dutch hospital (last 6 months)	12.5	14 (3.2)	2 (4.3)	1.34 (0.30–6.07)	
Proton pump inhibitor use (last 6 months)	14.1	55 (12.6)	9 (19.2)	1.64 (0.75–3.57)	
Antibiotic use					
Last 6 months	11.5	77 (17.7)	10 (21.3)	1.26 (0.60–2.64)	
Last 3 months	5.6	51 (11.7)	3 (6.4)	0.51 (0.15–1.71)	
Medication use (last 6 months)					
ADHD medication	40.0	3 (0.7)	2 (4.3)	**6.39 (1.04–39.23)**^**e**^	
Oral contraceptives	10.4	112 (25.8)	13 (27.7)	1.10 (0.56–2.16)	
Antidepressants	10.0	18 (4.1)	2 (4.3)	1.03 (0.23–4.57)	
Sleeping pills/tranquilizers	13.9	31 (7.1)	5 (10.6)	1.55 (0.57–4.19)	
Antihypertensive agents	13.6	19 (4.4)	3 (6.4)	1.49 (0.42–5.23)	
Statins	14.3	6 (1.4)	1 (2.1)	1.55 (0.18–13.16)	
Laxatives	10.0	9 (2.1)	1 (2.1)	1.03 (0.13–8.29)	
Stomach and/or bowel disease					
Acid reflux	16.7	35 (8.1)	7 (14.9)	2.00 (0.83–4.79)^d^	
Irritable bowel syndrome	8.8	52 (12.0)	5 (10.6)	0.88 (0.33–2.32)	
Crohn’s disease	50.0	2 (0.5)	2 (4.3)	**9.62 (1.32–69.96)**^**e**^	
Stomach and/or bowel complaints (last 4 weeks)	14.4	161 (37.0)	27 (57.5)	**2.30 (1.25–4.23)**^**e**^	2.18 (1.17–4.06)
*Leisure activities*					
Travel (last 6 months)					
No travel, travel to Western/Northern Europe, North America, Australia or New Zeeland	6.8	247 (56.8)	18 (38.3)	Ref	Ref
Travel to Southern/Eastern Europe	8.1	137 (31.5)	12 (25.5)	1.20 (0.56–2.57)	1.24 (0.58–2.66)
Travel to Africa, Asia or Latin America	25.0	51 (11.7)	17 (36.2)	**4.57 (2.21–9.47)**^**e**^	4.41 (2.11–9.19)
Swimming in fresh water (last 6 months)	9.0	183 (42.1)	18 (38.3)	0.86 (0.46–1.59)	
Swimming in salt water (last 6 months)	13.7	170 (39.1)	27 (57.5)	**2.10 (1.14–3.87)**^**e**^	
Used animal manure (last 6 months)	9.1	80 (19.4)	8 (19.5)	0.99 (0.44–2.23)	
*Diet and hygiene*					
Diet					
Vegetarian	4.8	20 (4.6)	1 (2.1)	0.47 (0.06–3.58)	
Non-vegetarian	9.6	403 (92.6)	43 (91.5)	Ref	
Pescatarian	20.0	12 (2.8)	3 (6.4)	2.34 (0.64–8.63)	
Hand washing frequency before food preparation					
(Almost) always	10.2	229 (52.6)	26 (55.3)	Ref	
Regularly/sometimes	8.7	179 (41.2)	17 (36.2)	0.84 (0.44–1.59)	
(Almost) never	12.9	27 (6.2)	4 (8.5)	1.31 (0.42–4.02)	
Hand washing frequency after toilet use					
(Almost) always	10.4	250 (57.5)	29 (61.7)	Ref	
Regularly/sometimes	8.6	170 (39.1)	16 (34.0)	0.81 (0.43–1.54)	
(Almost) never	11.8	15 (3.5)	2 (4.3)	1.15 (0.25–5.28)	
Uses dishcloth/scourer for more than 1 day	11.6	237 (54.5)	31 (66.0)	1.62 (0.86–3.05)^d^	

ADHD: Attention-deficit/hyperactivity disorder; CI: confidence interval; ESBL-E/K: extended-spectrum beta-lactamase or pAmpC-producing *Escherichia coli/Klebsiella pneumoniae*; IQR: interquartile range; OR: odds ratio; Ref: reference

^a^Including animal physiotherapists

^b^Including animal caretakers

^c^Weekly or more often

^d^*P* value < 0.20, considered in multivariable model

^e^*P* value < 0.05, indicated in bold, considered in multivariable model

### Longitudinal ESBL-E/K carriage

After a median of 6.3 months (range 5.7–8.5 months), 16/33 (48.5%) veterinary workers that were ESBL-E/K positive in the first sampling, tested positive again (Fig. 1). The same ESBL gene and *E. coli* ST combination was found in 14/16 (87.5%). This included one person carrying *bla*_CTX-M-15_ in a new *E. coli* ST at both sampling moments (with equal MLST results). Two persons were carrier of a different ESBL gene (also in different *E. coli* STs) at the second sampling moment (participant 8 and 14 in Additional file 2: Table S2). In the 6 months between the two faecal sampling moments these two participants did not travel to high prevalence countries, did not use antibiotics and were not admitted to a hospital.

### Household contacts and transmission of ESBL-E/K

Twenty-three household members distributed over 19 households of veterinary workers were included. Of the household members 30.4% were female and their median age was 36 years (min 30; max 56 years). They were partners of the veterinary workers (73.9%), or relatives (26.1%), and 91.3% used the same kitchen and bathroom. Other characteristics of household members are shown in Additional file 2: Table S3. Four household members (17.4%) were ESBL-E/K carrier (Fig. 1 and Table 4). The veterinary workers of three out of the four positive household members also tested positive during the second sampling. In these three pairs the gene *bla*_CTX-M-15_ was detected. This gene was located in the same *E. coli* ST in two pairs. The fourth positive household member carried the same ESBL gene and ST as the veterinary worker in the initial sampling round (*bla*_CTX-M-14_ on ST69).

**Table 4:** ESBL/pAmpC gene types, *E. coli* sequence types and general characteristics of household members

Characteristics	ESBL-E/K positive household members			
	1	2	3	4
Relation to VHW	Partner	Partner	Partner	Partner
Uses same kitchen/ bathroom as VHW	Yes	Yes	Yes	Yes
Hand washing after toilet use	(Almost) always	Sometimes	Sometimes	(Almost) always
Professional contact with animals	Yes, veterinary Assistant	Yes, hoof care (cows)	No	No
Health-care professional	No	No	No	No
Animal contact last 4 weeks	Dog, cat, rabbit, chicken	Dog, cat, cow	Dog, cat, mouse, horse	Dog
Hospitalization last 6 months	No	No	No	No
PPI use last 6 months	No	No	No	No
AB use last 6 months	No	No	No	No
Travel history last 6 months	Southern Europe	Western, Northern Europe	Western Asia	Southern Europe
ESBL/ pAmpC gene	*bla*_CTX-M-15_	*bla*_CTX-M-14_	*bla*_CTX-M-15_	*bla*_CTX-M-15_
*E. coli* ST	1193	69	69	6143
**ESBL-E/K results of corresponding veterinary healthcare worker**				
Results at T0				
ESBL/ pAmpC gene	*bla*_CTX-M-15_	*bla*_CTX-M-14_	*bla*_CTX-M-15_	*bla*_CTX-M-32_
*E. coli* ST	1193	69	131	68/48
Results at T1				
ESBL/ pAmpC gene	*bla*_CTX-M-15_	–	*bla*_CTX-M-15_	*bla*_CTX-M-15_
*E. coli* ST	1193	–	131	6143

AB: antibiotic; ESBL-E/K: extended-spectrum beta-lactamase or pAmpC-producing *Escherichia coli/Klebsiella pneumoniae*; pAmpC: plasmid-mediated AmpC; PPI: proton pump inhibitor; ST: sequence type; T0: first sampling moment; T1: second sampling moment; VHW: veterinary healthcare worker

## Discussion

In the present study among 482 veterinary healthcare workers from the Netherlands, the estimated prevalence of ESBL-E/K carriage of 9.8% (95%CI 7.4–12.7) was higher compared to the average prevalence of 5% in the Dutch population [4, 5]. An explanation for the higher prevalence found in the veterinary workers could be the frequent animal contact that (almost) all participants indicated.

We did not include a reference group as multiple large population-based studies have been performed in the last decade in the Netherlands [4, 5]. The largest Dutch general population study ESBLAT (ESBL-attribution analysis, performed Nov 2014–Nov 2016, n = 4177), that used culture methods comparable to our methods, found an adjusted prevalence for ESBL-producing Enterobacterales (excluding AmpC producers) of 5.0% (95% CI 3.4–6.1) [5]. This was significantly lower compared to the prevalence in veterinary workers after excluding the persons carrying AmpC producers, of 8.7% (42/482; 95% CI 6.5–11.6). Additional analyses were performed to compare these findings, taking into account potential differences between the study populations. When correcting for age, sex, country of birth, travel in the last 6 months, antibiotic use in the last 6 months and stomach and/or bowel complaints in the last 4 weeks, the risk of ESBL-E/K carriage in the veterinary workers was still significantly higher compared to the ESBLAT participants (OR 2.1; 95% CI 1.4–3.2). See Additional file 3 for a more detailed description of the analysis and results. The ESBLAT study was performed two years earlier, but there are no indications that the prevalence of ESBL-E carriage in the general Dutch population has significantly changed in this period. This indicates that working at a veterinary clinic and occupational exposure to animals might be the reason for the higher prevalence.

Multivariable analysis indicated traveling abroad to Africa, Asia and Latin America as the most important risk factor for ESBL-E/K carriage, which is an established factor in literature [17]. Recent stomach and bowel complaints was a novel predictor for ESBL-E carriage. Bowel complaints were recently also described as risk factor by Arcilla et al. in a group of travellers [18]. They found that pre-existing chronic bowel disease as well as traveller’s diarrhoea that persisted after return were important predictors for acquisition of ESBL-E during travel. Whether the recent stomach and bowel complaint among our participants were also linked to travel is unknown, although these complaints were more frequently registered by participants who travelled to Africa, Asia or Latin America (47% vs 38% in the rest of the participants, not statistically significant due to low numbers).

Our findings do not corroborate the results of two studies among Finnish and UK veterinary workers. In both countries a prevalence similar to the general population was reported [19, 20]. Compared to the Netherlands, carriage of ESBL/AmpC‐producing *E. coli* in Finnish livestock is low, with a prevalence of 0.8% in cattle, and up to 8.1% in broilers [21]. In combination with the ESBL-E/K prevalence of 6.8% in the population at large, working with livestock in Finland might not result in an increased risk of ESBL-E/K carriage [19]. In perspective, in the Netherlands 8.3% of dairy cow and 17.9% of broiler samples contained ESBL/AmpC-producing *E. coli* in the 2019 national surveillance [22]. For broilers this was already a tremendous decline from the prevalence of 66.0% observed in 2014. In the UK study, the prevalence in staff members and student in three veterinary hospitals was 6% (5/84) in a cross-sectional sample [20]. From a subgroup of participants that volunteered to provide additional samples, 25.9% (7/27) carried ESBL-producing *E. coli* at least once during a 6-week follow-up period. The low number of participants in the UK study made it hard to draw any conclusions from the logistic regression models and could have also impacted on the stability of the prevalence rates.

The prevalence of ESBL-E/K in companion animals and equines is not routinely monitored in the Netherlands, but according to recent studies the prevalence among horses, dogs and cats is 10.8%, 10.7% and 1.4%, respectively [11, 23]. A higher prevalence was found in diseased dogs and cats and in horses in a large equine clinic [7, 24]. Within the domestic setting or at the veterinary clinic, transmission of ESBL-E/K from animals to humans can occur [11, 25, 26]. The fact that most participants worked with multiple animal species (71% indicated contact with three or more species), could have hindered the investigation into the association with specific species. This was especially true for companion animals, since these practices are almost never solely devoted to one species. Furthermore, the percentage of participants working with specific livestock species was limited.

Although all ESBL genes that were found in the present study have also been demonstrated in animals, transmission between animals and the veterinary workers cannot be proven. In the present study, no sampling of animals visiting the clinics was performed. This was not considered since veterinary staff often has contact with numerous animals each day and long term carriage (≥ 6 months) is common among humans [27]. Therefore transmission could have occurred months before the sampling took place. Differences in the abundance of genes have been noted between animal species. In the Netherlands, in livestock *bla*_CTX-M-1_ and *bla*_CMY-2_ prevail [28], while in horses *bla*_CTX-M-1_ and *bla*_CTX-M-2_ are predominant [23, 24]. Among the veterinary workers *bla*_CTX-M-15_ was by far the most frequently found ESBL gene (53%). This gene is associated with transmission between humans or from the environment [28]. However, the proportion of *bla*_CTX-M-15_ has increased in Dutch veal calves and dairy cows in recent years [22]. Furthermore, in a previous study among domestic cats and dogs *bla*_CTX-M-15_ was the second most abundant gene type (after *bla*_CTX-M-1_) and human–dog co-carriage within households was demonstrated [11], although transmission from human to animal cannot be ruled out. In the present study almost 90% of the participants indicated occupational contact with dogs and more than 85% with cats. Furthermore, most participants had occupational contact with companion animals only (68.9%). Notably, *bla*_DHA-1_ was the third most abundant gene (n = 4; 8.5%), which was exclusively found in persons occupationally exposed to companion animals. This AmpC gene is not always screened for due to its low prevalence, but it has been detected in humans, pets, sheep and the environment, with *K. pneumoniae* as its favourite host [29]. The only ESBL-E/K positive *K. pneumoniae* that was found among the veterinary workers, harboured a *bla*_DHA-1_ gene. The predominant STs in this research do not correspond to animal *E. coli* and *K. pneumoniae* types, but to the types that were found in other recent studies on carriership in the general Dutch population [5, 28]. This does not necessarily mean that the source is also human, as it is known from studies in farmers and their animals that farmers often carry similar ESBL genes on similar plasmids as found in their animals, but in other *E. coli* types [8].

Almost half (48.5%) of the initially ESBL-E/K positive participants were still ESBL-E/K carriers 6 months later and 87.5% of these carried the same ESBL gene and strain combination, which indicates long term carriage. These rates were slightly higher compared with findings from a previous longitudinal study in the population at large (36.5% long-term carriers, 81.6% with identical ESBL-E isolates) [27]. In three out of four ESBL-E/K positive household pairs the same strain and gene was found, which could indicate transmission or exposure to the same source. However, to rigorously investigate transmission within the household the number of ESBL-E/K positive participants was limited. Furthermore, the time between the first sampling moment of the veterinary worker and the participation of the household member (approximately 6 months) might have been too long to assess transmission, since more than half of the veterinary workers positive at T0 were negative at T1.

## Conclusions

In this study the ESBL-E/K prevalence in a large group of veterinarians, veterinary technicians and veterinary assistants working with a wide variety of animal species in animal clinics throughout the Netherlands was investigated. The majority of participants worked in clinics for companion animals. Veterinary healthcare workers had a higher ESBL-E/K prevalence compared to the general Dutch population, which could not be explained by a higher occurrence of established risk factors such as antibiotic use and travel to countries with a high ESBL-E/K prevalence. Therefore, it seems plausible that occupational contact with animals in the animal healthcare setting is the reason for the higher prevalence, despite the absence of specific occupational risk factors. This, in combination with the occurrence of ESBL-E/K co-carriage within households, indicates that working in a veterinary clinic could be a source of introduction for ESBL-E/K into the general population, especially in countries with an ESBL-prevalence in animals that exceeds the prevalence in humans.

## Supplementary Information

**Additional file 1: Table S1.** Primers and PCR conditions used in this study. Overview of primers and conditions used in PCR screening and sequencing.

**Additional file 2: Tables S2.** ESBL/pAmpC gene types and *E. coli* sequence types in veterinary healthcare workers that were tested ESBL-E/K positive at both sampling moments (T0 and T1); **Table S3.** Characteristics of household members of ESBL-E/K positive veterinary healthcare workers.

**Additional file 3:** Comparison of veterinary healthcare workers with the general population. Results of the comparison of veterinary healthcare workers (AREND study) with the general population (ESBLAT study, Nov 2014–Nov 2016) using a multivariable logistic regression model (Table S4).

## Data Availability

The datasets used and/or analysed during the current study are available from the corresponding author on reasonable request.
