# Supplementary material for: ESBL/pAmpC-producing Escherichia coli and Klebsiella pneumoniae carriage among veterinary healthcare workers in the Netherlands
Source: Antimicrob Resist Infect Control. 2021 Oct 19;10:147. doi: 10.1186/s13756-021-01012-8 (PMC8524829; doi:10.1186/s13756-021-01012-8)
Supplement: Supplementary file 1 — Additional file 1: Table S1. Primers and PCR conditions used in this study. Overview of primers and conditions used in PCR screening and sequencing. [file 13756_2021_1012_MOESM1_ESM.docx]

**Additional file 1**

**Table S1. Primers and PCR conditions used in this study**

|  | **PCR name** | **Target** | **Primer name** | **Sequence (5′-3′)** | **Annealing temperature (°C)** | **Product size (bp)** | **Reference** |
| --- | --- | --- | --- | --- | --- | --- | --- |
| 1 | CTX-M group multiplex PCR | CTX-M group 1 | MultiCTXMGp1_for | TTAGGAARTGTGCCGCTGYA | 60 | 688 | 1 |
|  |  |  | MultiCTXMGp1-2_rev | CGATATCGTTGGTGGTRCCAT |  |  | 1 |
|  |  | CTX-M group 2 | MultiCTXMGp2_for | CGTTAACGGCACGATGAC | 60 | 404 | 1 |
|  |  |  | MultiCTXMGp1-2_rev | CGATATCGTTGGTGGTRCCAT |  |  | 1 |
|  |  | CTX-M group 9 | MultiCTXMGp9_for | TCAAGCCTGCCGATCTGGT | 60 | 561 | 1 |
|  |  |  | MultiCTXMGp9_rev | TGATTCTCGCCGCTGAAG |  |  | 1 |
| 2 | CTX-M-1 group sequence PCR | CTX-M group 1 | CTX-M-1g Fw | CCCATGGTTAAAAAATCACTGC | 62 | 944 | 2 |
|  |  |  | CTX-M-1g Rv | CAGCGCTTTTGCCGTCTAAG |  |  | 2 |
| 3 | CTX-M-2 group sequence PCR | CTX-M group 2 | CTX-M-2F | ATGATGACTCAGAGCATTCG | 56 | 884 | 3 |
|  |  |  | CTX-M-2R | TTATTGCATCAGAAACCGTG |  |  | 3 |
| 4 | CTX-M-9 group sequence PCR | CTX-M group 9 | CTX-M-9-1F | TGGTGACAAAGAGAGTGCAACG | 58 | 875 | 4 |
|  |  |  | CTX-M-9-4R | TCACAGCCCTTCGGCGAT |  |  | 4 |
| 5 | CTX-M-14/17 sequence PCR | CTX-M-14/17 | CTX-M-9_792F | CTATTTTACCCAGCCGCAAC | 55 | 238 | 5 |
|  |  |  | CTX-M-9_1029R | GTTATGGAGCCACGGTTGAT |  |  | 5 |
|  |  | CTX-M-14/17 | CTX-M-9_792F | CTATTTTACCCAGCCGCAAC | 55 | 426 | 5 |
|  |  |  | CTX-M-9_1217R | ATGGAAAAACAACCATGCGG |  |  | This study |
| 6 | TEM, SHV, OXA-1-like multiplex PCR | TEM | MultiTSO-T_for | CATTTCCGTGTCGCCCTTATTC | 60 | 800 | 1 |
|  |  |  | MultiTSO-T_rev | CGTTCATCCATAGTTGCCTGAC |  |  | 1 |
|  |  | SHV | MultiTSO-S_for | AGCCGCTTGAGCAAATTAAAC | 60 | 713 | 1 |
|  |  |  | MultiTSO-S_rev | ATCCCGCAGATAAATCACCAC |  |  | 1 |
|  |  | OXA-1-like | MultiTSO-O_for | GGCACCAGATTCAACTTTCAAG | 60 | 564 | 1 |
|  |  |  | MultiTSO-O_rev | GACCCCAAGTTTCCTGTAAGTG |  |  | 1 |
| 7 | TEM sequence PCR | TEM | TEM-F | GCGGAACCCCTATTTG | 58 | 964 | 6 |
|  |  |  | TEM-R | ACCAATGCTTAATCAGTGAG |  |  | 6 |
| 8 | SHV sequence PCR | SHV | OS5 | TTATCTCCCTGTTAGCCACC | 58 | 797 | 7 |
|  |  |  | OS6 | GATTTGCTGATTTCGCTCGG |  |  | 7 |
| 9 | CIT PCR | LAT-1 to LAT-3, BIL-1, CMY-2 to CMY-7, CMY-12 to CMY-18 and CMY-21 to CMY-23 | CIT_F | CGAAGAGGCAATGACCAGAC | 60 | 538 | 1 |
|  |  |  | CIT_R | ACGGACAGGGTTAGGATAGY |  |  | 1 |
| 10 | CMY sequence PCR | CMY group 2 | CMY-2-F | ATGATGAAAAAATCGTTATGCTGC | 58 | 1138 | 8 |
|  |  |  | CMY-2-R | GCTTTTCAAGAATGCGCCAGG |  |  | 9 |
| 11 | DHA sequence PCR-1 | DHA | DHA_-52F | GTGAATCTGACGATACTTGC | 58 | 717 | 10 |
|  |  |  | DHA_666R | TAACCGTACGCATACTGGC |  |  | 10 |
| 12 | DHA sequence PCR-2 | DHA | DHA_263F | TCACAGGTGTGCTGGGTG | 58 | 905 | 10 |
|  |  |  | DHA_+27R | TCCGCAGGGGCCTGTTCAG |  |  | 10 |
| 13 | ACC sequence PCR-1 | ACC | ACC_-68F | GCATGCTGATTGGCGTGC | 56 | 1289 | 10 |
|  |  |  | ACC_+79R | AGGGCGTGCTGTAATACC |  |  | 10 |
| 14 | ACC sequence PCR-2 | AAC | ACC_118F | CAGCCGCTGATGCAGAAG | 56 | 668 | 10 |
|  |  |  | ACC_785R | CCCCATATTGGCTTGCAC |  |  | 10 |
| 15 | ACT sequence PCR | ACT | ACT_-77F | CACAGTCAAATCCAACAGAC | 56 | 1242 | 10 |
|  |  |  | ACT-MIR_+19R | AGCGCCACCCGGCAATG |  |  | 10 |

The screening for the presence of ESBL/AmpC genes consisted of three steps:

Step 1. All isolates were screened for the presence of CTX-M, TEM, SHV, OXA and CIT-AmpC genes by primer sets 1, 6 and 9.

Step 2. All isolates with a negative result in step 1 were tested by disk diffusion test.

Step 3. All isolates with an AmpC phenotype in step 2 were screened for DHA, ACC and ACT using primer sets 11-15.

All other primer sets were used for sequencing.

**References**

1. Dallenne C, Da Costa A, Decré D, Favier C, Arlet G. Development of a set of multiplex PCR assays for the detection of genes encoding important β-lactamases in Enterobacteriaceae. *J Antimicrob Chemother* 2010; **65**: 490-95.

2. Carattoli A, García-Fernández A, Varesi P, et al. Molecular epidemiology of Escherichia coli producing extended-spectrum β-lactamases isolated in Rome, Italy. *J Clin Microbiol* 2008; **46**: 103-08.

3. Steward CD, Rasheed JK, Hubert SK, et al. Characterization of clinical isolates of Klebsiella pneumoniae from 19 laboratories using the National Committee for Clinical Laboratory Standards extended-spectrum β-lactamase detection methods. *J Clin Microbiol* 2001; **39**: 2864-72.

4. Paauw A, Fluit AC, Verhoef J, Leverstein-van Hall MA. Enterobacter cloacae outbreak and emergence of quinolone resistance gene in Dutch hospital. *Emerg Infect Dis* 2006; **12**: 807-12.

5. Dierikx CM, van Duijkeren E, Schoormans AH, et al. Occurrence and characteristics of extended-spectrum-β-lactamase- and AmpC-producing clinical isolates derived from companion animals and horses. *J Antimicrob Chemother* 2012; **67**: 1368-74.

6. Olesen I, Hasman H, Aerestrup FM. Prevalence of β-lactamases among ampicillin-resistant Escherichia coli and Salmonella isolated from food animals in Denmark. *Microb Drug Resist* 2004; **10**:334-40.

7. Arlet G, Rouveau M, Philippon A. Substitution of alanine for aspartate at position 179 in the SHV-6 extended-spectrum β-lactamase. *FEMS Microbiol Lett* 1997; **152**: 163-67.

8. Kruger T, Szabo D, Keddy KH, et al. Infections with nontyphoidal Salmonella species producing TEM-63 or a novel TEM enzyme, TEM-131, in South Africa. *Antimicrob Agents Chemother* 2004; **48**: 4263-70.

9. Hasman H, Mevius D, Veldman K, Olesen I, Aerestrup FM. β-Lactamases among extended-spectrum β-lactamase (ESBL)-resistant Salmonella from poultry, poultry products and human patients in The Netherlands. *J Antimicrob Chemother* 2005; **56**:115-21.

10. van Hoek AHAM, Schouls L, van Santen MG, Florijn A, de Greeff SC, van Duijkeren E. Molecular Characteristics of Extended-Spectrum Cephalosporin-Resistant Enterobacteriaceae from Humans in the Community. *PLoS One* 2015; **10**: e0129085.
