# Supplementary material for: ESBL/pAmpC-producing Escherichia coli and Klebsiella pneumoniae carriage among veterinary healthcare workers in the Netherlands
Source: Antimicrob Resist Infect Control. 2021 Oct 19;10:147. doi: 10.1186/s13756-021-01012-8 (PMC8524829; doi:10.1186/s13756-021-01012-8)
Supplement: Supplementary file 2 — Additional file 2: Tables S2. ESBL/pAmpC gene types and E. coli sequence types in veterinary healthcare workers that were tested ESBL-E/K positive at both sampling moments (T0 and T1); Table S3. Characteristics of household members of ESBL-E/K positive veterinary healthcare workers. [file 13756_2021_1012_MOESM2_ESM.docx]

**Additional file 2**

**Table S2. ESBL/pAmpC gene types and *E. coli* sequence types in veterinary healthcare workers that were tested ESBL-E/K positive at both sampling moments (T0 and T1)**

|  | **T0** | | **T1** | |
| --- | --- | --- | --- | --- |
| **Subject** | **ESBL/pAmpC gene** | **ST** | **ESBL/pAmpC gene** | **ST** |
| 1 | *bla*_CTX-M-15_ | 1193 | *bla*_CTX-M-15_ | 1193 |
| 2 | *bla*_CTX-M-15_ | new^a^ | *bla*_CTX-M-15_ | new^a^ |
| 3 | *bla*_CTX-M-15_ | 131 | *bla*_CTX-M-15_ | 131 |
| 4 | *bla*_DHA-1_ | 10 | *bla*_DHA-1_ | 10 |
| 5 | *bla*_CTX-M-15_ | 131 | *bla*_CTX-M-15_ | 131 |
| 6 | *bla*_CTX-M-1_ | 349 | *bla*_CTX-M-1_ | 349 |
| 7 | *bla*_CTX-M-1_ | 69 | *bla*_CTX-M-1_ | 69 |
| 8 | *bla*_CTX-M-15_ | 656 | *bla*_CTX-M-55_ | 167 |
| 9 | *bla*_CTX-M-14_ | 38 | *bla*_CTX-M-14_ | 38 |
| 10 | *bla*_CTX-M-14_ | 38 | *bla*_CTX-M-14_ | 38 |
| 11 | *bla*_CTX-M-15_ | 131 | *bla*_CTX-M-15_ | 131 |
| 12 | *bla*_CTX-M-15_ | 10 | *bla*_CTX-M-15_ | 10 and 2689 |
| 13 | *bla*_DHA-1_ | 349 | *bla*_DHA-1_ | 349 |
| 14 | *bla*_CTX-M-32_ | 68 and 48 | *bla*_CTX-M-15_ | 6143 |
| 15 | *bla*_CTX-M-15_ | 405 | *bla*_CTX-M-15_ | 405 |
| 16 | *bla*_CTX-M-15_ | 131 | *bla*_CTX-M-15_ | 131 |

ESBL: extended-spectrum beta-lactamase; pAmpC: plasmid-mediated AmpC; ST: sequence type; T0: first sampling moment; T1: second sampling moment.

^a^ Both sequence types are the same.

**Table S3. Characteristics of household members of ESBL-E/K positive veterinary healthcare workers**

|  | **Household members**  **n = 23** | |
| --- | --- | --- |
| **Determinant** | **ESBL-E/K negatives**  **n = 19** | **ESBL-E/K positives**  **n = 4** |
|  | **n (%)** | **n (%)** |
| Gender |  |  |
| male | 13 (68.4) | 3 (75.0) |
| female | 6 (31.6) | 1 (25.0) |
| Age, years (median (min-max)) | 34 (28-56) | 43.5 (34-58) |
| Urbanisation level |  |  |
| very high | 3 (15.8) | 0 (0) |
| high/moderate | 9 (47.4) | 2 (50.0) |
| low/very low | 7 (36.8) | 2 (50.0) |
| Born in the Netherlands | 17 (89.5) | 3 (75.0) |
| Relation to VHW |  |  |
| partner | 13 (68.4) | 4 (100) |
| relative | 6 (31.6) | 0 (0) |
| Uses same kitchen as VHW | 17 (89.5) | 4 (100) |
| Diet |  |  |
| vegetarian | 1 (5.3) | 0 (0) |
| non-vegetarian | 18 (94.7) | 4 (100) |
| pescatarian | 0 (0) | 0 (0) |
| Uses same bathroom as VHW | 17 (89.5) | 4 (100) |
| Hand washing frequency after toilet use |  |  |
| always/usually | 11 (57.9) | 2 (50.0) |
| regularly/sometimes | 8 (42.1) | 2 (50.0) |
| rarely/never | 0 (0) | 0 (0) |
| Profession with animal contact | 3 (15.8) | 2 (50.0) |
| Healthcare professional | 1 (5.3) | 0 (0) |
| Animal contact, last 4 weeks | 19 (100) | 4 (100) |
| dogs | 18 (94.7) | 4 (100) |
| cats | 14 (73.7) | 3 (75.0) |
| rabbits/Guinea pigs/hamsters | 7 (36.8) | 1 (25.0) |
| rats/mice | 1 (5.3) | 1 (25.0) |
| birds | 3 (15.8) | 0 (0) |
| cattle | 1 (5.3) | 1 (25.0) |
| sheep | 3 (15.8) | 0 (0) |
| goats | 3 (15.8) | 0 (0) |
| chicken | 2 (10.5) | 1 (25.0) |
| other poultry | 0 (0) | 0 (0) |
| pigs | 0 (0) | 0 (0) |
| horses | 5 (26.3) | 1 (25.0) |
| Hospitalized in Dutch hospital, last 6 months | 1 (5.3) | 0 (0) |
| Proton pump inhibitor use last 6 months | 0 (0) | 0 (0) |
| Antibiotic use |  |  |
| last 6 months | 2 (10.5) | 0 (0) |
| last 3 months | 1 (5.3) | 0 (0) |
| Travel, last 6 months |  |  |
| no travel, travel to Western/Northern Europe, North America, Australia or New Zeeland | 12 (63.2) | 1 (25.0) |
| travel to Southern/Eastern Europe | 2 (10.5) | 2 (50.0) |
| travel to Africa, Asia or Latin America | 5 (26.3) | 1 (25.0) |
| Swimming in fresh water, last 6 months | 3 (15.8) | 1 (25.0) |
| Swimming in salt water, last 6 months | 6 (31.6) | 1 (25.0) |
| Used animal manure, last 6 months | 5 (26.3) | 1 (25.0) |

ESBL-E/K: extended-spectrum beta-lactamase or pAmpC-producing *Escherichia coli/Klebsiella pneumoniae*; VHW, veterinary healthcare worker.
