# Supplementary material for: ESBL/pAmpC-producing Escherichia coli and Klebsiella pneumoniae carriage among veterinary healthcare workers in the Netherlands
Source: Antimicrob Resist Infect Control. 2021 Oct 19;10:147. doi: 10.1186/s13756-021-01012-8 (PMC8524829; doi:10.1186/s13756-021-01012-8)
Supplement: Supplementary file 3 — Additional file 3: Comparison of veterinary healthcare workers with the general population. Results of the comparison of veterinary healthcare workers (AREND study) with the general population (ESBLAT study, Nov 2014–Nov 2016) using a multivariable logistic regression model (Table S4). [file 13756_2021_1012_MOESM3_ESM.docx]

**Additional file 3**

**Comparison of veterinary healthcare workers with the general population**

The veterinary healthcare workers (AREND study) were compared to the general population (ESBLAT study, Nov 2014 – Nov 2016) [1] using a multivariable logistic regression model including participants from both studies. In order to quantify the risk difference a ‘study’ variable (AREND versus ESBLAT) was included, as well as potential risk factors for ESBL-E/K carriage that were queried in both studies. These included age, gender, country of birth, travel in the last 6 months, antibiotic use in the last 6 months and stomach and/or bowel complaints in the last 4 weeks. A number of differences have been taken into account in the comparison:

- In the ESBLAT study 2 persons were carrier of an ESBL-producing *E. cloacae*, these persons were not included as ESBL-E/K positives in the comparison.
- The pAmpC positive persons from the AREND study (n=5) were not included as ESBL-E/K positives in the comparison.
- The age limit was set to 18 years, resulting in the exclusion of 572 children that participated in the ESBLAT study.

Finally, 482 veterinary healthcare workers (prevalence 8.7%; 42/482; 95% CI 6.5-11.6) and 3605 persons from the general population (prevalence 4.5%; 163/3605; 95% CI 3.9-5.2) were included in the analysis.

The results of the analysis are shown in the table below. When corrected for differences between the two study populations, the risk of ESBL-E/K carriage (excluding pAmpC) in the veterinary healthcare workers was still significantly higher compared to the general population of the ESBLAT study (OR 2.1; 95% CI 1.4-3.2).

**Table S4. Comparison between veterinary healthcare workers (AREND study) and the general population (ESBLAT study) by multivariable logistic regression analysis**

|  | **AREND** | | **ESBLAT** | | **Adjusted OR (95%CI)** |
| --- | --- | --- | --- | --- | --- |
|  | **n** | **%** | **n** | **%** |  |
| Study (AREND vs ESBLAT) | 482 | 100 | 3605 | 100 | 2.12 (1.40-3.19) |
| Gender |  |  |  |  |  |
| Male | 73 | 15.2 | 1606 | 44.6 | 1.46 (1.06-2.02) |
| Female | 409 | 84.9 | 1995 | 55.3 | Ref. |
| Age (years) |  |  |  |  | 1.00 (0.99-1.02) |
| 18-30 | 116 | 24.1 | 267 | 7.4 | - |
| 31-40 | 176 | 36.5 | 316 | 8.8 | - |
| 41-50 | 93 | 19.3 | 599 | 16.6 | - |
| 51-60 | 73 | 15.2 | 867 | 24.1 | - |
| 61-70 | 24 | 5.0 | 977 | 27.1 | - |
| 71-80 | 0 | 0 | 470 | 13.0 | - |
| 81-90 | 0 | 0 | 100 | 2.8 | - |
| 91-100 | 0 | 0 | 5 | 0.1 | - |
| Country of birth |  |  |  |  |  |
| Netherlands | 471 | 97.7 | 3424 | 95.0 | Ref. |
| other | 11 | 2.3 | 132 | 3.7 | 1.96 (1.02-3.77) |
| Travel during last 6 months |  |  |  |  |  |
| no travel, travel to Western/Northern Europe, North America, Australia or New Zeeland | 265 | 55.0 | 2309 | 64.1 | Ref. |
| travel to Southern/Eastern Europe | 149 | 30.9 | 901 | 25.0 | 1.01 (0.70-1.46) |
| travel to Africa, Asia or Latin America | 68 | 14.1 | 308 | 8.5 | 3.05 (2.08-4.48) |
| Antibiotic use last 6 months | 87 | 18.1 | 405 | 11.2 | 1.35 (0.90-2.02) |
| Stomach and/or bowel complaints last 4 weeks | 177 | 36.7 | 776 | 21.5 | 1.40 (1.00-1.98) |

AREND: antibiotic resistant bacteria in Dutch veterinarians study; CI: confidence interval; ESBLAT: ESBL-attribution analysis study; OR: odds ratio.

**References**

1. van den Bunt G, van Pelt W, Hidalgo L, Scharringa J, de Greeff SC, Schürch AC, et al. Prevalence, risk factors and genetic characterisation of extended-spectrum beta-lactamase and carbapenemase-producing Enterobacteriaceae (ESBL-E and CPE): a community-based cross-sectional study, the Netherlands, 2014 to 2016. Euro Surveill. 2019;24(41):1800594.
